# Editorial: Social capital and immigrant wellbeing in the digital age

**DOI:** 10.3389/fsoc.2024.1524996

**Published:** 2024-11-29

**Authors:** Adekunle Adedeji, Franka Metzner, Johanna Buchcik

**Affiliations:** ^1^Department of Social Work, Hamburg University of Applied Sciences, Hamburg, Germany; ^2^Department of Health Sciences, Hamburg University of Applied Sciences, Hamburg, Germany; ^3^Bremen International Graduate School of Social Sciences, Constructor University, Bremen, Germany; ^4^Department of Medical Psychology, University Medical Center Hamburg-Eppendorf, Hamburg, Germany

**Keywords:** social capital, integration, migration, digital age, globalization

The rapid evolution of digital technology has transformed the ways immigrants interact, integrate, and sustain wellbeing in host societies. In particular, social capital, both in traditional and digital forms, has proven essential to immigrants' ability to navigate challenges related to cultural adaptation, mental health, and economic stability. This Research Topic, “*Social capital and immigrant wellbeing in the digital age*,” explores the complex intersections of digital tools, social networks, and cultural adjustment, highlighting the dual role of digitalization in enhancing and sometimes complicating immigrant wellbeing.

The digital era brings both opportunities and new stressors for immigrant populations, as seen in Zhang et al.'s study on digital financial inclusion among migrant workers in China. In this paper, the authors explore how digital finance—through its reach and accessibility—mitigates overwork, an issue pervasive among rural-to-urban migrant workers. Digital financial inclusion positively impacts job quality and reduces excessive labor by expanding access to savings and loan products that enable financial stability. However, this alleviation is not uniform, and disparities in digital access persist across different regions and worker demographics. The authors argue for targeted financial policies that promote equitable digital access, particularly in under-resourced communities, to ensure that the benefits of digital finance reach all migrant groups.

Another dimension of social capital's digital impact is evident in Joji and Mapaling's research on mental health help-seeking among Indian immigrant youth in South Africa. Immigrant youth, often caught between their home and host cultures, face unique pressures, with many viewing mental health help-seeking through a culturally filtered lens that may discourage support. Joji and Mapaling highlight the role of digital and social media as both facilitators and barriers to mental health support. Social media offers platforms for discussion, raising awareness and accessibility, yet cultural stigmas and family pressures still limit its effectiveness. The study underscores the importance of culturally sensitive mental health interventions utilizing digital platforms to create safe open dialogue and support spaces.

Further examining the sociocultural predictors of immigrant wellbeing, Shekriladze and Javakhishvili investigate the roles of cultural distance, language fluency, and discrimination in shaping psychological outcomes among Georgian immigrants in Europe. They find that host culture engagement and language proficiency strongly predict wellbeing, underscoring the importance of supportive sociocultural policies. While cultural integration is beneficial, their research suggests that immigrants' perceived discrimination and lack of language fluency may hinder adjustment, especially in contexts with significant intercultural distance. These findings echo the importance of inclusive language and anti-discrimination policies in fostering immigrant adaptation and reducing acculturative stress.

The digital age's impact on immigrant family dynamics, particularly among children, emerges as a critical factor in Wang et al.'s exploration of cyberbullying among left-behind children (LBC) in China. Their study reveals that children separated from their parents due to migration are more susceptible to cyberbullying, a modern risk amplified by unsupervised internet access. Parent-child communication, often disrupted by physical distance, is shown to be a protective factor against cyberbullying. Wang et al. argue that enhancing digital literacy and promoting family communication can mitigate cyber risks, emphasizing the need for parental involvement in monitoring online activities, especially for children in vulnerable family situations.

Collectively, these studies reflect the multifaceted role of digitalization in immigrant wellbeing. Digital tools and platforms provide critical resources, bridging physical gaps and fostering connection. Yet, they also introduce new risks that can exacerbate vulnerability, particularly among youth and economically disadvantaged migrant groups. These findings underline the need for a comprehensive approach to social capital that extends beyond traditional social networks to include digital forms of interaction, which are increasingly essential in contemporary immigrant life.

In a broader context, the articles within this Research Topic suggest actionable insights for policymakers and practitioners. First, digital equity must be prioritized to ensure that immigrant communities, particularly those in low-income or remote areas, can access digital tools that support economic stability, mental health, and social inclusion. Second, culturally nuanced mental health interventions should leverage digital platforms, creating accessible, stigma-free resources tailored to specific immigrant groups. Finally, policies promoting language acquisition and anti-discrimination are vital in creating welcoming environments that enable immigrants to build meaningful social connections.

As digital technology continues to shape the immigrant experience, future research should focus on the long-term implications of digital inclusion on immigrant wellbeing, exploring both the benefits and potential psychological or social trade-offs. Additionally, the role of social capital within digital platforms, such as social media groups and online communities, warrants further exploration as a potential source of resilience and support for immigrants in navigating their host societies.

In conclusion, the digital age redefines social capital for immigrant populations, offering a complex mix of empowerment and challenge. The studies presented in this Research Topic highlight the opportunities digital tools provide and the nuanced, often unexpected obstacles they pose. As immigrants navigate the dual landscapes of digital and physical worlds, ensuring equitable access and culturally relevant support systems remains essential to fostering immigrant wellbeing in a globally interconnected society.

